# Mental health services in Egypt during the COVID-19 pandemic

**DOI:** 10.1186/s43045-022-00270-5

**Published:** 2022-12-22

**Authors:** Tarek Okasha, Menan Rabie, Nermine Mahmoud Shaker, Nesreen Mohsen, Mahmoud EL-Habiby, Dina Aly El-Gabry, Eman Gaber, Nemat Ali, Mohamad Ali, Maha Sayed

**Affiliations:** 1 Neuropsychiatry Department, Okasha Institute of Psychiatry, Ain Shams University, Cairo, 1156 Egypt; 2 The General Secretariat of Mental Health and Addiction Treatment (GSMHAT), Cairo, Egypt

## Abstract

**Background:**

The COVID-19 pandemic has irreversibly altered the medical landscape. Compromised mental health among medical staff and the general population has called for new patient approaches, therapies, and medical services, among which Telehealth features prominently.

**Results:**

This paper discusses the structure, approach, and efficiencies of pandemic-related mental health services in Egypt and summarizes responses and initiatives launched by the government of Egypt. A particular focus on the General Secretariat of Mental Health and Addiction Treatment (GSMHAT)’s action plan as well as Ain Shams University’s hospital Okasha Institute of Psychiatry sheds light on localized responses to the pandemic’s psychological impact.

**Conclusions:**

Data showcasing the several types of Telehealth employed are used to derive conclusions about the merits and challenges of emerging online treatments within the context of COVID-19.

## Background

The outbreak of the COVID-19 virus has aroused enormous global attention [1]. Rapidly transmitted in late January 2020, the pandemic has shaken the globe in more ways than one, affecting individuals, families, and communities on several levels [2]. Mental health suffered in particular: loss, bereavement, isolation, uncertain prognoses, looming severe shortages of resources for testing and treatment for protecting responders and health care providers from infection profoundly affected individuals. The imposition of unfamiliar public health measures that infringe on personal freedom, large and growing financial losses, and conflicting messages from authorities contributed to widespread emotional distress and increased risk for psychiatric illness associated with COVID-19 [3].

### The COVID-19 pandemic in Egypt

In Egypt, over 800 cases were confirmed by the beginning of April 2020, reaching 6465 within one month as well as 430 fatality cases, causing anxiety and fear in the general population [4, 5]. The government of Egypt established a unique care model under the World Health Organization’s (WHO) supervision, assigning specific hospitals as “quarantine hospitals” for COVID-19 patients. Operational medical teams reside in the hospital for 14 days continuously and, following a negative test for SARS-CoV-2, are then released for self-isolation at home for another 14 days. Positive-swab doctors (if any) are admitted to the same quarantine hospital to receive medical care [6]. Egypt also announced the extension of curfews across the country and the suspension of international air passenger arrivals, maintained effective contact tracing with proper quarantine mechanisms, and expanded the number of peripheral laboratories capable of testing for COVID-19 [7].

### Psychological impact of the pandemic on the Egyptian population

Confirmed or suspected cases of COVID-19 suffered from great psychological pressure, other health-related problems, and fear of severe disease consequences and the contagion [1, 8]. The limited understanding of COVID-19 and its outbreak in combination with continuously overwhelming news lead to anxiety and fear in the public [9]. Consequently, individuals experienced loneliness, denial, anxiety, depression, insomnia, and despair, which may have lowered treatment adherence and increased risk of aggression and suicide. Suspected isolated cases developed obsessive-compulsive symptoms, such as repeated temperature checking and disinfection. Furthermore, strict quarantine and mandatory contact tracing policies by health authorities are conducive to societal rejection, financial loss, discrimination, and stigmatization. The public at large also experienced boredom, disappointment, and irritability under the isolation measures [10]. Health professionals are of no exception, as they have a duty of care for infected patients, are in close contacts with patients’ families and relatives, and sometimes face public enquiry [8].

In Egypt, Arafa et al.’s [11] study on citizens in 4 Egyptian governorates in 2020 reported a high prevalence of depression (67.1%), mild to moderate (44.6%), and severe to very severe depression (22.5%). They found anxiety and stress to be prevalent, with 30.6% mild to moderate anxiety and 22.9% severe to very severe, as well as 33.8% mild to moderate stress and 15.0% severe to very severe. Another study using the Impact Event scale showed that 41.4% of 510 adults felt horrified and helpless, while 338 (66.3%) felt apprehensive [12]. The numbers of infected and deceased physicians and other health care workers were extremely high compared to the general population, with a mortality rate 16%. Many suffered from mental health problems, including stress, anxiety, depression, and burnout symptoms, particularly female healthcare workers, young-age staff with less experience, and medical staff working in intensive care units, fever, and quarantine hospitals [4, 6, 13–16].

## National coordination group for mental health and psychosocial response for COVID-19 pandemic in Egypt

The General Secretariat of Mental Health and Addiction Treatment (GSMHAT) is a governmental body affiliated to the Ministry of Health and Population, dedicated to the provision of mental health services and substance use treatment and rehabilitation through inpatient psychiatric hospitals, outpatient mental health care centers, and primary health care services. GSMHAT supervises 19 governmental mental health hospitals across Egypt and works as the main educational body in mental health and addiction treatment, providing training to its own employees as well as all other mental health service providers.

**Fig. 1:**
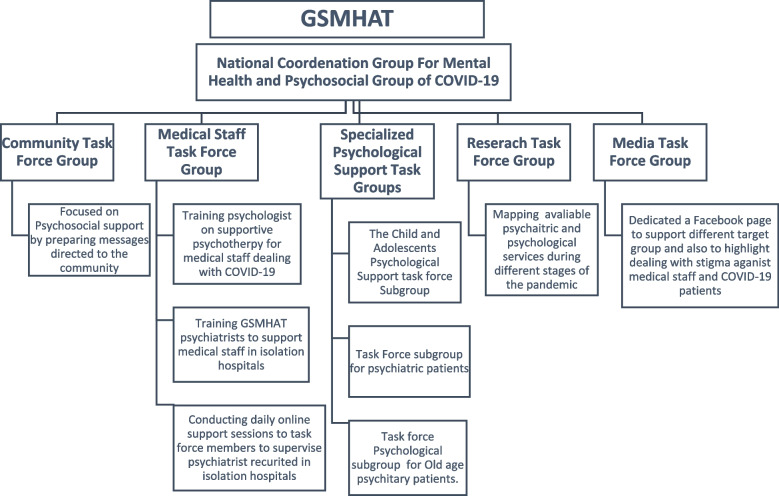
GSMHAT task force groups during COVID-19 pandemic

At the outset of COVID-19, GSMHAT perused the available data necessary to launch an action plan for mental health during the pandemic. With the support of the WHO, it addressed the MHPSS (Mental Health Psychosocial Support) aspects of the COVID-19 outbreak in Egypt for all persons living in the country, including refugees. In March 2020, GSMHAT launched a media campaign and the national coordination group for mental health and psychosocial response, integrating the MHPSS to understand mental health and psychosocial issues and improving the coordination among governmental and non-governmental sectors. It invited ministries, agencies, and organizations working on MHPSS in Egypt to liaise with the coordination group. Five task force groups were formed that facilitated a rapid and coordinate response to specific and/or priority areas identified in the emergency as seen in Fig. 1.

*The Community Task Force Group* focused on psychosocial support, preparing messages on how to overcome pandemic-related stressors and improve mental health and wellbeing, training staff, and setting up an emergency hotline.

The *Medical Staff Task Force Group* trained psychologists on supportive psychotherapy for medical staff dealing with COVID-19 patients, training GSMHAT psychiatrists to support medical staff and patients with psychiatric problems in quarantine hospitals, and conducting daily online support sessions for task force members to supervise psychiatrists.

The *Specialized Psychological Support Task Force Group* included task force subgroups for children and adolescents, psychiatric patients, and old age.

*The Child Psychological Support Task Force Subgroup* developed a guideline manual for dealing with children in quarantine, hospitals, and at home, as well as manuals for autistic children and children with intellectual disability.

The *Task Force Subgroup for Psychiatric Patients* offered medications in outpatient clinics for old cases covering 3 months, emergency-only evaluation of new cases, and phone call consultations including follow-up availability. Psych education videos for OCD, autism, and anxiety disorders patients were performed online.

The *Research Task Force Group* assessed the needs of medical teams and the community, mapped available psychosocial and psychiatric services during different stages of the pandemic, researched multidisciplinary research priorities for COVID-19, and covered research ethics in emergency situations.

The *Media Task Force Group* dedicated a Facebook page to support COVID-19 patients and their relatives, elderly people, those with children during quarantine, and medical staff.

### The hotline service extension

In April 2020, GSMHAT extended the existing hotline services to provide psychological support to all segments of society on a 24/7 basis (Table 1). Forty-four percent of those seeking consultations complained of anxiety symptoms, 22% complained of depressive symptoms, and 15% complained of insomnia. Those requiring further interventions were referred to the nearest GSMHAT hospital.

**Table 1:** Data related to hotline service during the COVID 19 pandemic

		April	May	June	July	August
Total no of calls		1047	891	886	533	484
Calls seeking psychological support related to COVID-19		52.8%	56.1%	54%	39%	22%
		*N* = 553	*N* = 500	*N* = 478	*N* = 207	*N* = 106
New cases		100%	79%	87%	82%	84%
Follow-up cases		-	21%	13%	18%	16%
Males		40%	47%	47%	39%	38%
Females		60%	53%	53%	61%	62%
Age group	Adults	90%	92%	93%	94%	91%
	Below 18 years	2.5%	4%	3%	3%	6%
	Over 60 years	3.5%	4%	4%	3%	3%
Calls needed supervision/higher consultation		15%	24%	19%	25%	18%
Cases receiving psychosocial support	General community	100%	79%	68%	65%	77%
	COVID-19 cases	-	11%	18%	26%	14%
	Contacts to COVID-19 cases	-	8%	10%	7%	5%
	Medical staff working in quarantine hospitals	-	1%	2%	-	2%
	Medical staff working in general hospitals	-	1%	2%	2%	2%
Need for medications		30%	37%	39%	40%	55%
Referral to	GSMHAT hospitals	-	63%	76%	89%	82%
	University hospitals	-	1%	3%	3%	2%
	Online group psychotherapy	-	22%	18%	8%	15%
	Both online group psychotherapy and GSMHAT hospitals	-	12%	3%	-	1%

In May 2020, follow-up services including online therapy sessions were added: suicidal cases, medical staff dealing with COVID-19 patients and suffering from stress and work-related psychological symptoms, COVID-19 diagnosis, and COVID-19 patients in quarantine either at home or in quarantine hospitals.

In June 2020, 52% of cases seeking psychosocial support (*N* = 260) needed a follow-up call once, 23% needed it twice, 8% 3 times, another 8% 4 times, 4% 7 times, and another 4% 8 times. Nineteen percent needed higher consultation due to needing more in-depth psychological intervention or being at risk for self-harm or suicide. UNICEF’s Department of Child Protection prompted GSMHAT to provide psychological counseling by phone for children.

In August 2020, consultations seeking psychological support from cases below 18 years increased to 6% (3% male and 3% female adolescents).

## Developing a manual for the least hazardous medications for neuropsychiatric symptoms with COVID-19

A committee of psychiatrists and pharmacists affiliated with GSMHAT prepared a manual for minimally hazardous medications for neuropsychiatric symptoms with COVID-19. It included psychotropic medications, usable with minimal side effects such as psychiatric symptoms and drug interactions with medications for COVID-19.

## Liaison psychiatric services inside the quarantine hospitals

In April 2020, a specialized team of psychiatrists affiliated to GSMHAT hospitals was recruited to follow-up on cases which required psychological support or had psychiatric symptoms in 15 quarantine hospitals across Egypt. They provided psychological support to COVID-19 cases and medical staff for 14 days in rotation, assessing referred cases, and devising treatment plans for each case. In addition, they raised medical staff’s awareness about the expected psychological impact of admission to quarantine hospitals on COVID-19 patients. Online training courses were offered for health care providers in quarantine hospitals to improve coping strategies and stress management skills among medical staff, including crisis intervention and psychological debriefing.

From April till August 2020, 672 cases were referred to the psychiatrists recruited in quarantine hospitals. 88.7% (*n* = 596) were COVID-19 patients from the community, 7.4% (*n* = 50) were medical staff infected with COVID-19 during their work, and 3.9% (*n* = 26) were medical staff who needed psychological support during their work in quarantine hospitals. Eighty-three percent of cases were aged from 18 to 60, 18% were aged above 60, and 2% were aged below 18. Males and females were equally represented (39.3%, 39.1%, respectively), and 2.1% were children and adolescents. Seventy-six percent had a positive history of previous psychiatric disorder. Most cases complained of bad moods, sleep difficulty, refusal of food or medication, fear, severe anxiety, nervousness, and irritability. Forty-five percent of cases were prescribed medications, 92% received psychoeducation and psychological support, 6% received CBT, 1% received behavioral modification therapy, and 1% received story telling therapy. An appropriate treatment plan approved by medical staff prescribed medications to patients. Obstacles and limitations facing psychiatrists in quarantine hospitals included difficulty in dealing with critical cases in ICU, difficulty with completing case data at the time of assessment, and follow-ups, as the presence of any tools was prohibited by infection control measures. To our knowledge, this was a unique service applied in Egypt during the pandemic.

## A specialized hospital for COVID-19 psychiatric patients (Al Fatimia Cairo Hospital)

In June 2020, the head of Ministry of Health and Population allocated Al Fatimia Cairo Hospital as the first to admit psychiatric patients infected with COVID-19. A team of psychiatrists treated those patients during their quarantine. Eighteen cases were admitted during the first wave of the pandemic, and another 16 cases were admitted during the second wave. Fifty-six percent of patients were aged above 60 years, and 44% aged from 18 to 60 years. Sixty-nine percent were males, and 31% were females. Only 6% had relatives infected with COVID-19. Sixty-three percent had positive history of medical illness, and 69% had positive history of psychiatric illness. Ninety-four percent received definite diagnosis according to DSM-5, and only 6% received preliminary diagnosis due to short stay, deterioration of the medical condition, or uncooperativeness of the family to provide psychiatric history. Forty-five percent were diagnosed with schizophrenia, 22% were diagnosed with bipolar disorder, 22% were diagnosed with dementia, and 11% were diagnosed with intellectual disability. Eighty-seven percent were prescribed psychotropic medications, 55% received psychological support, 30% received psychoeducation, 10% received cognitive behavioral therapy, and 5% received behavioral modification.

## Okasha Institute of Psychiatry’s psychiatric service for the community

Home-based telehealth rather than in-person services dominated globally during the pandemic [17]. Ain Shams University hospitals pioneered the use of Telemedicine services in Africa and Middle East years before COVID and expanded its scope and quality during the pandemic. The University’s hospital, the Okasha Institute of Psychiatry, launched a flexible and renewable plan to be adjusted easily according to changing COVID-19 restrictions, delivering telepsychiatry by approaching clients through smart phones. It covered all psychiatric consultations within the inpatient wards, using an online/phone call format to comply with the strict infection control restrictions, especially in quarantine hospitals with the evolution of the psychiatric symptoms in COVID patients. The institute’s plan to cover the mental health effects of the pandemic covered 4 themes: (1) effects of COVID infection on mental health state for infected subjects, (2) mental health problems facing medical care providers, (3) effects of quarantine and social distances on the mentally ill patients and how their conditions were affected by massive environmental changes, and (4) assessment of service provided by the Institute during the pandemic.

By mid-April 2020, the institute provided a WhatsApp Triage service, where a trained consultant received messages about clients’ inquiries and reply within 2 h. The application’s main function was to determine the nature and severity of the mental health problem, decide which service response would best cater to the needs of the patient, and assess the urgency of the required response. This included risk assessment based on medical history, determining whether patients were at risk of harming themselves or others as a result of their mental state, and to assess other risks related to mental illness. The clinician assigned a category of urgency to the case, ranging from “extreme risk” to “low risk”: (a) video consultation for more data collection and mental state examination or (b) E.R. at the Okasha Institute of Psychiatry for proper assessment and management in risky cases (Table 2).

**Table 2:** Demonstration for WhatsApp consultations, Okasha Institute of Psychiatry

WhatsApp triage consultations		
	First month	Second month
Total consultations	*N* = 87	*N* = 500
Males	49.5 % (*N* = 43)	34% (*N* = 170)
Females	50.5% (*N* = 44)	66% (*N* = 330)
COVID-19 related consultations (anxiety or sleep)	None	2.8 % (*N* = 14)
Referral to E.R.	3.5% (*N* = 3)	2% (*N* = 10)
Referral to psychotherapy online sessions	13.8% (*N* = 12)	15% (*N* = 75)
Referral to video consultation	9.2% (*N* = 8)	1.8% (*N* = 9)
Follow-up cases	5.8% (*N* = 5)	10% (*N* = 50)
Referral to other specialties	4.6% (*N* = 4)	2.4% (*N* = 12)

To counteract pandemic-related mental health repercussions, the Okasha Institute of Psychiatry shifted all substance abuse patients to online clinics. The Ain Shams University virtual hospital official platform was launched in mid-June, 2020. General adult psychiatry as well as online addiction clinic was introduced as well. The child and adolescent psychiatry unit launched the service with a weekly child clinic and a weekly adolescent clinic, which were directed by a senior consultant for guaranteeing proper service. After 2 weeks of starting the service, there was a total of 51 reservations, most of them in the general adult psychiatry clinics. Only 21 video consultations were successful; the remaining reservations malfunctioned due to technical problems (e.g., improper Internet signal), difficulties for clients in dealing with the site, and no-shows.

Announcements on the official Facebook page of Ain Shams University’s virtual hospital, posters in all clinic rooms, and the E.R. and reception desk clerks informed clients about these new services. By the end of 2021, the virtual psychiatry clinics received 2327 bookings with 920 successful consultations, a 39.5% success rate. This percentage may appear weak, yet when highlighting the challenges facing the service, it was satisfactory. Lack of access to technology, Internet, insufficient technology infrastructure, and lack of training for patients and providers marked common barriers to telehealth implementation. Potential for liability, cost to purchase and maintain equipment, patient/parent reluctance to participate, and patient/parent comfort with technology were frequently noted.

## Conclusions

Egypt has set a wonderful example in facing the challenges related to mental health services caused by COVID-19 pandemic, making it a leading example in Africa and the Middle East.

## Data Availability

All generated or analyzed data during this study are included in the published work.

## Abbreviations

COVID-19: Coronavirus disease of 2019
CBT: Cognitive behavioral therapy
E.R: Emergency room
GSMHAT: General Secretariat of Mental Health and Addiction Treatment
ICU: Intensive care units
MHPSS: Mental Health Psychosocial Support
OCD: Obsessive compulsive disorder
SARS-CoV-2: Severe acute respiratory syndrome coronavirus 2
UNICEF: The United Nations Children’s Fund
WHO: World Health Organization
